# Multiple jejunal perforation secondary to intestinal tuberculosis infection: a case report

**DOI:** 10.11604/pamj.2017.27.78.11061

**Published:** 2017-06-02

**Authors:** Anthony Lyonga Ngonge, Domin Sone Majunda Ekaney, Carlson-Babila Sama, Joffi Musonge-Effoe, Valery Sammah Effoe, Gerald Ekwen

**Affiliations:** 1 Faculty of Health Sciences, University of Buea, Buea, Cameroon; 2 Department of Surgery, Baptist Hospital Mutengene, Cameroon; 3 Bambalang Medicalised Health Centre, Northwest Cameroon and Galactic Corps Research Group(GCRG), Buea, Cameroon; 4 Buea Regional Hospital, Buea, Southwest Cameroon; 5 Department of General Internal Medicine, Morehouse School of Medicine, Atlanta, Georgia, USA

**Keywords:** Abdominal tuberculosis, intestinal perforation, HIV infection

## Abstract

Abdominal tuberculosis (TB) may affect any part of the gastrointestinal tract resulting in significant morbidity and mortality. There is an increase in the incidence of abdominal TB favored by the emergence of multi-drug resistant Mycobacterium tuberculosis and immunosuppression especially from HIV co-infection. Our case is that of a 31 year old HIV-positive woman, adherent to antiretroviral therapy, who presented with a 2 month history of progressive abdominal distention, drenching night sweat and fatigue, but without fever. She was admitted on a presumptive diagnosis of peritoneal TB, and suddenly developed signs and symptoms of an acute abdomen. Laboratory investigations showed a CD4+ count of 155 cells/µL, white blood cell count of 15,700 cells/mm^3^ and haemoglobin of 8.0g/dl. An emergency laparotomy revealed small bowel caseous necrosis with multiple jejunal perforations. Ziehl-Nelsen staining of operative specimen was positive for acid fast bacilli. Given her immunodeficiency status, clinical signs and symptoms, CD4 cell count > 50 cells/µL, and intestinal sample showing caseous necrosis and perforations, a final diagnosis of intestinal TB was made. In conclusion, abdominal tuberculosis may mimic a number of intra-abdominal pathologies; thus should always be considered as a differential diagnosis in patients presenting with acute abdomen in TB-endemic areas especially in an HIV-positive individual.

## Introduction

Tuberculosis (TB), one of the dreaded tropical diseases of poverty till date, still poses a great burden to health care systems around the globe especially in Sub-Saharan Africa. In 2013, it accounted for about 1.4 million deaths globally with two-third of these deaths occurring in resource-limited countries [1]. Among communicable diseases, it is second only to HIV infection as a leading cause of death [2]. TB has the potential to affect any organ-system in the body leading to either the pulmonary or extra-pulmonary forms. The rising incidence of multi-drug resistant strains of Mycobacterium tuberculosis and HIV co-infection has led to an upswing in the incidence and severity of both forms of the disease [3, 4]. Abdominal TB, is the sixth most common site of extra-pulmonary involvement and can affect any part of the digestive system including the liver, spleen or pancreas with the most common site being the ileocecal region [5]. Abdominal TB is usually secondary to either pulmonary infection or from a primary infection of an adjacent extra-thoracic organ such as spine or kidneys. The bacteria may reach the abdomen either via hematogenous route, contiguous spread from infected lymph nodes or by swallowing-up of infected sputum [5]. Despite recent innovations in the practice of medicine, abdominal TB still poses a major diagnostic challenge especially in resource-limited countries, primarily due to the unavailability of many diagnostic tools. Secondly it presents with non-specific symptoms, thus mimicking a wide variety of other intra-abdominal pathologies such as acute appendicitis, Chron's disease, peritoneal carcinomatosis [6]. Isolated multiple simultaneous jejunal perforations secondary to TB infection is an uncommon finding even in TB endemic regions. In this report, we describe such an uncommon constellation.

## Patient and observation

A 31-year old Sub-Saharan African woman known with HIV infection for the past 3 years and compliant to antiretroviral treatment and cotrimoxazole prophylaxis presented to our emergency unit with a 2-month history of slowly progressive abdominal distention, drenching night sweat and generalized fatigue without fever. She had regular bowel movement, no abdominal pain and no weight loss. She had two prior hospital visits for the same symptoms during which she received medical care but had no favorable outcome. On physical examination, she had a temperature of 37.4°C, blood pressure of 100/62 mmHg, heart rate of 93 beats/min and respiratory rate of 19 cycles/min. She was mildly icteric. Abdominal exam was remarkable for distention with a positive fluid thrill and shifting dullness, mild right hypochondriac tenderness without rebound tenderness and no guarding. Her chest exam and cardiovascular as well as the rest of the examination was unremarkable. Initial laboratory findings included a normal white cell count level of 8900 cells/mm^3^ (Normal Value (Nv): 4000-10000cells/mm^3^). Haemoglobin (HB) was 10g/dl (Nv 12-15g/dl). CD4+ count of 155 cells/µL, elevated aspartate amino transferase (AST) 97 IU/L (Nv; < 40U/L) and alanine amino transferase (ALT) 83IU/L (Nv: < 50IU/L). HBsAg and Anti-HCV tests were negative. Serum Creatinine was 1.2mg/dl (Nv: 0.6-1.2mg/dl). Abdominal ultrasound revealed massive ascites and a normal liver architecture. Chest X-ray film did not show any lung field changes and sputum analysis for acid fast bacilli were negative. A presumptive diagnosis of peritoneal TB based on a history of HIV infection, night sweats, generalised fatigue and ascites was made with a differential diagnosis of hepatitis based on elevated liver transaminases, jaundice and right hypochondriac tenderness. Initial management constituted of a diagnostic and therapeutic paracentesis with drainage of 2 liters of straw- coloured fluid. She was placed on albumin infusion and Amoxicillin 500mg TID empirically prior to ascitic tap. Analysis of peritoneal ascitic fluid revealed a white cell count of 30 cells/µL with lymphocytic predominance. Ziehl-Neelsen staining of the centrifuged aspirate was negative for acid fast bacilli. Biochemistry analysis of the fluid was not done due to a lack of reagents in our center. A PPD skin test done during admission read 14mm induration in favour of TB infection.

**Figure 1:**
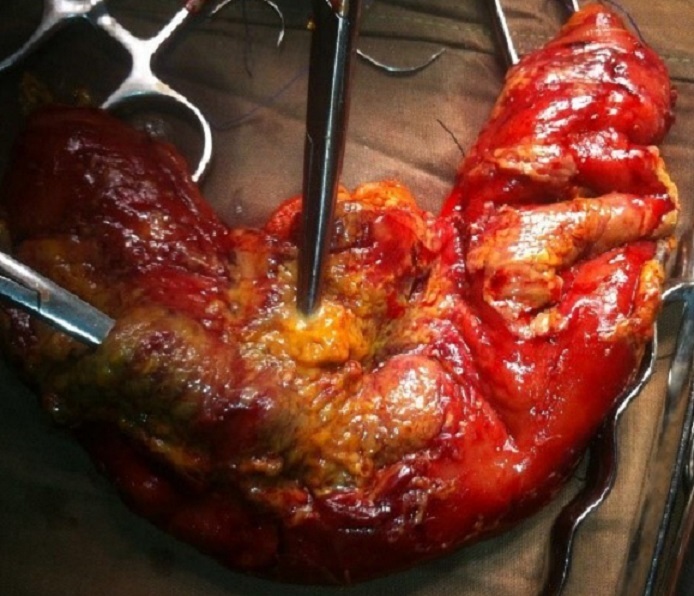
Caseous necrosis of the jejunum with multiple perforation sites

The plan of management for TB was discussed with the patient, but she declined treatment. The patient requested to be discharged from the hospital, despite advice from the treating physicians not to. She signed to leave against medical advice. Two days after discharge, she was readmitted in the hospital for generalized abdominal pain with rebound tenderness, spontaneous vomiting, low-grade fever of 38.8°C, blood pressure (BP) of 70/40 mmHg, weak thready pulse at 115 beats/min and cold clammy skin. Serum Creatinine was 3.5mg/dl, BUN of 78mg/dl (NV: 7-18mg/dl). A repeat white cell count showed leukocytosis of 15,700 cells/mm^3^ with neutrophil predominance of 87%. She had haemoglobin of 8g/dl. A presumptive diagnosis of spontaneous bacterial peritonitis was made. Initial management included 2 liters of intravenous normal saline 0.9% infusion, metronidazole 500mg, ampicillin 1g administered intravenously and transfusion of 2 units of cross- matched O Rhesus positive whole blood pre-operatively.Emergency laparotomy via a midline incision revealed about 4 liters of purulent peritoneal fluid, multiple enlarged peritoneal nodes measuring about 0.5 to 2mm thick, caseous necrosis of the omentum, and small bowel wall with three distinct sites of jejunal perforations (Figure 1). Resection of the necrotic jejunal segment with a primary end-to-end anastomosis was done. The intraoperative period was complicated by a cardiac arrest which was managed with cardiopulmonary resuscitation (CPR), clamping of the abdominal aorta along with administration of IV adrenaline and oxygen support. A sample of the purulent material was sent for laboratory analysis which showed a high load of acid fast bacilli on Ziehl-Neelsen staining. During the post-operative period, she was placed on parenteral nutrition, intravenous fluids, metronidazole 500mg 8hourly and Ampicillin 1g 6 hourly via the intravenous route. She was restricted from enteral feeding except for per os administration of anti-tuberculosis medication for the first 3 postoperative days. She had a favourable evolution with return of bowel movements and resumed oral feeding on post-operative day 4 against medical advice. On post-operative day 8, the patient developed increasing abdominal pains, associated with constipation and mild abdominal distention. Enteral feeding was again restricted. By post-operative day 10, her bowel sounds were absent and abdominal exam was remarkable for rebound tenderness and guarding. A repeat emergency laparotomy revealed a breakdown of her anastomosis. A repair was successfully done. Parenteral nutrition and antibiotics were continued. Two days after her second laparotomy, the patient had a cardiopulmonary arrest. Resuscitation efforts were unsuccessful.

## Discussion

The key clinical feature in this case as evident from the patient's history was the delay in arriving at an accurate diagnosis of abdominal TB which consequently led to a delay in the initiation of anti-TB treatment. The initial differential diagnosis on admission of hepatitis were misguided by the elevated liver transaminases associated with massive ascites in addition to a negative acid fast bacilli stain of the peritoneal fluid. However our history and physical examination on admission enabled us to gear our investigations towards the work-up of the possibility of an underlying abdominal TB infection. Her PPD skin test result of 14mm (normal value < 10mm or 5mm for immune-competent and immunosuppressed patients respectively), despite the negative test result from Ziehl- Neelsen staining of her peritoneal aspirate, enabled us to establish a presumptive diagnosis of abdominal TB. Extra pulmonary tuberculosis, though not very common among immune-competent individuals has been seen to be very common in immunosuppressed persons (20% vs 50% respectively), while 25-30% presents with concomitant pulmonary and extra-pulmonary TB [6, 7]. There incidence in Sub-Saharan Africa may even be higher than reported due to under-diagnosis and hence underreporting of such cases. Data from the World Health Organisation (WHO) shows that, the burden of TB especially in resource-limited settings is high. In 2014, WHO statistics from Africa showed a TB incidence of 2,700, 000 and a TB mortality of 450,000 compared to Europe which had a TB incidence of 340,000 with a TB mortality of 33,000. These differences can be attributable to poverty, poor hygiene standards, overcrowding, inaccessibility to heath facilities, unavailability of modern diagnostic tools in most health facilities and stock-out in the supply of medications to most regions in Africa. Three types of abdominal TB have been described in the literature depending on its presentation: 1) ascitic type, which presents with massive accumulation of peritoneal fluid along with multiple peritoneal nodules of 1-2mm thickness. 2) dry type which presents with omental thickening, adhesions to small bowel loops especially the terminal ileum which sometimes lead to inflammation, perforation or scarring of the bowel wall resulting in intestinal obstruction. 3) glandular type which presents with varying degrees of mesenteric lymph node enlargement which are hard in consistency and less mobile. This form also presents with scant to moderate amounts of ascites [8]. Our patient's intra-operative findings were consistent with a mixture of both the ascitic and dry types described above as evident by the massive purulent ascites, inflammation with multiple perforations of the jejunum along with multiple enlarged peritoneal lymph nodes. A number of investigations could have aided in the diagnosis of abdominal TB in our case, though none of them is 100% sensitive. The gold standard is to culture the mycobacterium from specimens (sputum, pleural and peritoneal fluid) in a Lowenstien-Jensen medium followed by PCR which were both not available in our health facility [9, 10].

However Ziehl-Neelson staining of the peritoneal fluid on admission was negative. This concurs with reports from other studies which showed that staining of peritoneal fluid is positive only in less than 3% of specimens [9]. Analysis of the purulent fluid obtained intra-operatively with Ziehl-Neelson stain showed a high load of acid fast bacilli. Further analysis such as serum- ascitic albumin gradient (SAAG), serum-ascitic protein gradient and imaging (CT scan) were not done due to limited resources at our disposal. However, despite the negative findings on chest exam, sputum analysis and chest X-ray, her PPD skin test results of 14mm coupled with her medical history were strongly suggestive of TB infection. A negative sputum test and negative chest X-ray findings do not rule out TB as concomitant pulmonary and abdominal TB only occurs in less than 25% of patients [5]. There are several case reports similar to ours, which describes abdominal TB resulting in an acute abdomen [11]. These clinical cases are similar to that of our patient by the presence of ascites, omental thickening with caseous necrosis, terminal ileal or gastric perforation. However, none of these patients were reported to have multiple sites of jejunal perforation. Due to limited resources in our setting, we were unable to pursue further work-up for our patient as indicated. As a result, a good and detailed clinical history and physical exam along with a high index of suspicion aided in our diagnosis. This is true for other settings [12]. The analysis of the purulent intra-operative peritoneal fluid which revealed numerous acid fast bacilli was key to our diagnosis and management of this patient. We therefore strongly reiterate on the routine collection of intra-operative specimen as good surgical practice. A peritoneal biopsy done earlier in the management of our patient could have helped provide a diagnosis earlier and improve on the outcome. We were unable to culture our sample for isolation of Mycobacterium tuberculosis to make a definitive diagnosis. However, given the patient's immunodeficiency status, clinical signs and symptoms, caseous necrosis observed on operative sample, and CD4 cell count > 50/µL, intestinal TB was the most likely diagnosis. Other nontuberculous Mycobacteria intestinal infections are possible in HIV patients, such as disseminated Mycobacterium avium complex (D-MAC); however these generally occur at a CD4 cell count < 0/µL. The mainstay in the management of abdominal TB similar to pulmonary TB is with oral anti-TB medications for a period of at least 6 months or more depending on the individual case. Strict follow-up of patients to ensure both compliance to treatment and evaluation for adverse drug effect is of paramount importance to ensure favorable outcome. Also, HIV- positive patients who are already on Anti-retroviral (ARV) drugs are encouraged to continue with both anti-TB and ARV drug protocols. Whereas those not on ARVs with CD4+ count < 50cells/ul are usually initiated on ARV medication two weeks post initiation of anti-TB treatment and within 8weeks for those with CD4+ count > 50cells/ul in order to reduce the frequency and severity of the occurrence of immune reconstitution syndrome [13]. Surgical intervention is usually reserved for patients who present with complications correctable by surgery such as intestinal perforation just as in our case, intestinal obstruction, or stricture [8].

## Conclusion

Abdominal TB, though not a very common finding in developing countries, has been on the rise owing to co-infection with HIV/AIDS. Its diagnosis especially in resource-limited settings could be very challenging. Thus a thorough clinical history including history of sick contacts, HIV status, good clinical exam with a high index of suspicion is paramount to an early diagnosis and thus early initiation of anti-TB treatment which may improve outcomes of the disease.

## Competing interests

The authors declare no competing interests.
